# Bond Behavior of Reinforced Concrete Considering Freeze–Thaw Cycles and Corrosion of Stirrups

**DOI:** 10.3390/ma14164732

**Published:** 2021-08-22

**Authors:** Shuo Liu, Maohua Du, Yubin Tian, Xuanang Wang, Guorui Sun

**Affiliations:** 1 Key Lab of Structures Dynamic Behavior and Control of the Ministry of Education, Harbin Institute of Technology, Harbin 150090, China; 2019221009@chd.edu.cn (S.L.); hitwangxuanang@163.com (X.W.); 19B933012@stu.hit.edu.cn (G.S.); 2 School of Highway, Chang’an University, Xi’an 710064, China; 3 Ordnance Engineering College, Naval University of Engineering, Wuhan 430033, China; 18207157778@139.com; 4 Key Lab of Smart Prevention and Mitigation of Civil Engineering Disasters of the Ministry of Industry and Information Technology, Harbin Institute of Technology, Harbin 150090, China; 5 School of Civil Engineering, Central South University, Changsha 410075, China

**Keywords:** bond behavior, reinforced concrete, freeze–thaw cycle, stirrup corrosion, numerical simulation

## Abstract

In relatively cold environments, the combination of freeze–thaw and steel bar corrosion is a key factor affecting the durability of concrete. The adjustment of the stirrup ratio would change the mechanical performance of surrounding concrete, while the circumferential compressive stress can further improve the bonding performance. Hence, based on eccentrically tensioned specimens, the influence of corrosion of stirrups and freeze–thaw of concrete on bond properties is discussed in this paper. The monotonic pull-out test of reinforced concrete specimens is carried out to study the variation rules of bond strength and slip between steel bar and concrete under the coupling action of corrosion rate, freeze–thaw times and stirrup spacing. Based on the experimental data, the empirical formula for the ultimate bond strength is obtained, and a bond–slip constitutive model is established considering the stirrup spacing, stirrup corrosion rate and freeze–thaw times. Then, a refined finite element pull-out specimen model is established by ABAQUS simulation, and the numerical simulation results are compared with the real test ones, so as to make up for the deficiencies in the test and lay the foundation for further finite element analysis.

## 1. Introduction

The degradation of reinforced concrete (RC) structures due to freeze–thaw cycles and reinforcement corrosion is a major problem that must be faced in infrastructure construction [1,2,3,4], causing the reduction of the service life of reinforced concrete structures and the increase in maintenance costs. The main hazard of freeze–thaw cycles is that they can reduce the basic mechanical and deformation properties of concrete, thus affecting the durability and bearing capacity of RC structures [5,6,7]. The corrosion of reinforcement consumes the original steel and produces expansive corrosion products, leading to a reduction in the area and deterioration of the mechanical properties of the reinforcement [8,9,10]. In addition, the corrosion of reinforcement would form a barrier layer at the interface between reinforcement and concrete, which can reduce the bonding properties between reinforcement and concrete [11,12,13]. In view of the importance of these factors, it is necessary to further study the bond behavior of RC structures considering freeze–thaw cycles and corrosion of stirrups.

At present, many scholars have conducted research on the durability of reinforced concrete after freeze–thaw cycles. Yu et al. [6] established the life prediction and cumulative model of concrete considering the effects of freeze–thaw cycles based on the experimental research to better predict the natural life of concrete under the actual freeze–thaw environment. Hamze et al. [14] made a concrete analysis of a dam in Canada that had been renovated three times due to freeze–thaw damage and assessed the key location where the structure was most vulnerable to frost damage. Ma et al. [15] conducted a series of freeze–thaw cycles and bond–slip tests and concluded that the bond performance of concrete with severe freeze–thaw damage deteriorated rapidly under the continuous load. According to the pull-out test data for three types of deformed rebars, Ji et al. [16] obtained the bond stress–slip curves after freeze–thaw and put forward the empirical formula of peak bond strength considering the effect of freeze–thaw cycles. On the basis of the existing bond–slip model, Wang et al. [17] proposed a new bond–slip model considering the freeze–thaw damage effect on concrete and applied it to the fiber model. These studies not only explore the effects of freeze–thaw cycles on bond–slip behavior but also further promote the application of reinforced concrete in the engineering field.

In addition, many researchers have conducted considerable studies on the corrosion of steel reinforcement and the durability of concrete. Bhargava et al. [3] proposed a simple empirical model to evaluate the reduction of bond strength under steel bar corrosion in RC structures. Bai et al. [18] proposed an empirical model of bond strength and a simplified bond–slip constitutive equation that considers the influence of reinforcement corrosion and temperature exposure. Almusalam et al. [19] prepared the calibration curve and established the relationship between the duration of current and the corrosive degree, they concluded that the ultimate bond strength between steel and concrete increased at the initial stage of corrosion. Fan et al. [20,21,22] applied distributed fiber optic sensors to investigate the corrosion-induced three-stage concrete cracking process. With the development of concrete cracks, the corrosion of the steel reinforcement becomes serious and the bond strength is greatly reduced. Sanz et al. [23] carried out push-out tests on concrete prisms reinforced with smooth steel tubes in order to study the bond loss caused by the corrosion of steel bar. Fang et al. [9] carried out the pull-out tests on smooth and deformed steel bars with stirrups or not, and found that corrosion had no substantial effect on the bond strength of constrained deformed steel bars. In terms of research on the effect of corrosion on reinforcement, in addition to main reinforcement corrosion receiving attention, stirrup corrosion has also received concern. Through the monotonic slip loading test, Zhou et al. [24] found that the bond strength of specimens with 15% mass loss of stirrups was lower than that of specimens without stirrups. Two more years passed, Zhou et al. [25] further investigated the effect of stirrup corrosion on the bond performance, and the results showed that the effect of stirrup corrosion on bond strength was less than main reinforcement corrosion. Lin et al. [26] discovered that compared to the case that merely main reinforcement was corroded, coupled corrosion of the main reinforcement and stirrups exacerbated bond deterioration. Moodi et al. [27] concluded that the stirrup corrosion had the greatest effect on the enhancement of the bond strength, ductility index and energy dissipation at 25% corrosion level by four-point bending test and response surface method.

In reality, the bond performance of RC structures is affected by many factors, and the coupling effect of freeze–thaw cycles and reinforcement corrosion is very common. However, most of the previous studies focused on the influence of a single factor, and less on the interaction of steel corrosion and freeze–thaw cycles. Therefore, it is necessary to study the coupling effect of freeze–thaw and steel corrosion. In addition, most tests adopt the center tensioned specimens, but the center tension approach tends to ignore the effect of stirrups, while the change of stirrup fitting rate can change the mechanical properties of the surrounding concrete, and the circumferential compressive stresses generated by the stirrups can enhance the bond performance. The eccentric pull-out test can fully consider the influence of stirrups, and this form of stress is very common in RC structures in actual engineering. However, up to now, there are few studies on eccentric tension tests, so it is necessary to conduct this form of test to make up for the lack of existing studies.

To solve the above problems, 21 reinforced concrete prismatic specimens were fabricated and subjected to freeze–thaw cycle tests for different periods. Then, the constant current corrosion method was used to corrode the stirrups, and finally, the eccentric tension test was carried out. Based on the experiment, the bond–slip behavior of RC prism specimens under the coupling action of three factors (steel bar corrosion, freeze–thaw cycle and stirrup ratio) was studied. At the same time, the bond–slip constitutive relationship between steel and concrete after freeze–thaw cycles and stirrup corrosion was explored. Moreover, the finite element analysis (FEA) was further performed, and the test results were analyzed and compared with the simulation data, verifying its rationality and applicability.

## 2. Test Setup and Procedure

### 2.1. Design and Fabrication of Specimens

At present, the research on bonding is mainly based on center tension and beam test. The test in this paper involves the freezing and thawing conditions of concrete. Due to the size limitation of the testing machine, the conditions of the testing machine could not meet the requirements of beam specimens, so the eccentric tension test is adopted.

According to ASTM C900-19 [28], the specimens were prisms with a size of 100 × 100 × 300 mm^3^, and the HRB400 longitudinal-ribbed steel bars with a diameter of 20 mm were embedded at the corresponding position. The configuration of the stirrup ring is a rectangular frame with a length and width of 70 mm formed by round steel bars with a diameter of 8 mm. In this test, parts without stirrups are marked as 0, and parts with a stirrup spacing of 100 mm and 150 mm are marked as 100 and 150 respectively. Moreover, two PVC pipes with a length of 50 mm are embedded at the stress measurement position and the beam end of the specimens, respectively. A foaming agent was filled between the steel bar and PVC to prevent cement intrusion during pouring and eliminate the local squeezing effect of the pressure-bearing steel plate at the end of the specimens. Moreover, the embedded pipes could prevent the stress concentration at the applied end which made the average bond strength of the specimens higher and avoided data distortion.

**Figure 1:**
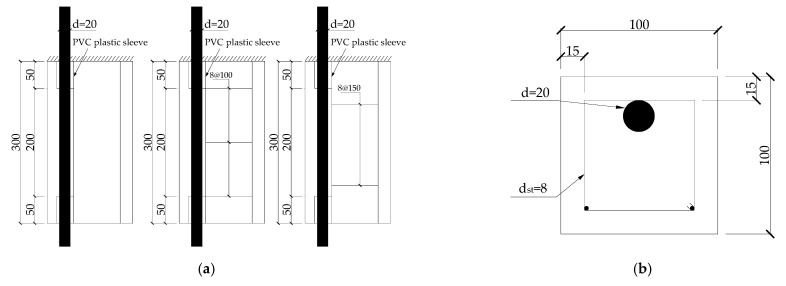
Test specimen (mm): (**a**) Different stirrup arrangement; (**b**) Cross-section front.

The yield strength of longitudinal reinforcement and smooth stirrups is 481 MPa and 334 MPa respectively, and the corresponding elastic modulus is 198 GPa and 213 GPa, respectively. The volume of concrete required for this test is about 0.1 m^3^, it was tested in the laboratory. The axial compressive strength of concrete used in specimens is 43.5 MPa, and the water–cement ratio is 0.37. The mix proportion of concrete is water:cement:sand:stone = 160:427:763:1014. The specimen label is uniformly expressed as Cs-ω-n, and the corresponding specific meaning is the stirrup spacing-corrosion percentage of the stirrups number of freeze–thaw cycles. Figure 1 shows the dimensions of specimens, and the different test cases are shown in Table 1.

### 2.2. Freeze–Thaw Scheme for Specimens

**Figure 2:**
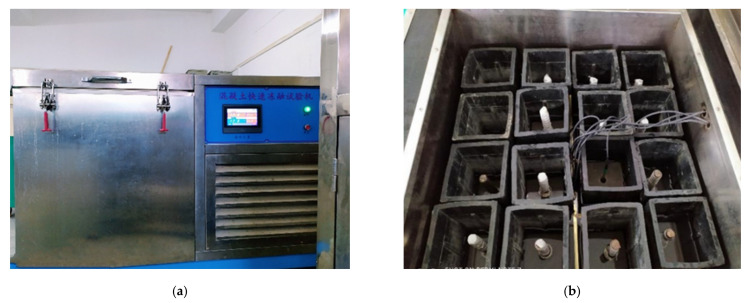
Freeze–thaw method: (**a**) Instrument; (**b**) Internal configuration.

The concrete was cured for 28 days under standard conditions. Before freezing and thawing, the specimens were fully immersed for 3 days, and then they were frozen and thawed in a rapid freezing and thawing instrument. The test instrument could freeze and thaw up to 15 specimens at one time, and the test instrument is shown in Figure 2. The freezing liquid was injected into the quick freeze–thaw apparatus before the test started, and the water was prevented from being mixed into the freezing liquid. In this experiment, the time of a freeze–thaw cycle was controlled within 2–4 h, and the number of freeze–thaw cycles was 15 times and 30 times, respectively. The freezing and thawing temperatures were kept at (−20~−18) °C and (18~20) °C, respectively. After the end of 15 cycles, the relevant specimens were taken out, and the empty space was filled with non-test specimens to ensure the overall temperature balance in the testing machine.

**Figure 3:**
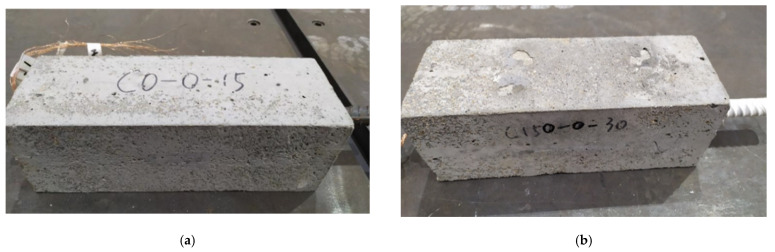
A freeze–thaw specimen: (**a**) After 15 freeze–thaw cycles; (**b**) After 30 freeze–thaw cycles.

In the freeze–thaw test, it is inevitable that the concrete would be damaged by freezing and thawing. Therefore, the appearance of the specimens taken out from the freeze–thaw machine reflects the degree of freeze–thaw damage to the specimens. Figure 3 shows the appearance of each batch of concrete specimens after experiencing 15 and 30 freeze–thaw cycles. As can be seen from Figure 3, the concrete surface did not change significantly after 15 freeze–thaw cycles, while some cracks and defects appeared on the concrete surface after 30 freeze–thaw cycles. The mass of the specimens before and after the freeze–thaw cycle was measured to further obtain the mass loss rate based on the ratio of the change value to the initial mass. Moreover, the relative dynamic elastic modulus of the specimens was measured by the ratio of the square of the transverse fundamental frequency of the specimens after and before the freeze–thaw cycles. It is found that after 15 and 30 freeze–thaw cycles, the average mass loss rates are 0.32% and 0.68%, respectively, and the average dynamic elastic modulus are reduced by 1.24% and 3.21%, respectively.

### 2.3. Corrosion Scheme for Specimens

**Figure 4:**
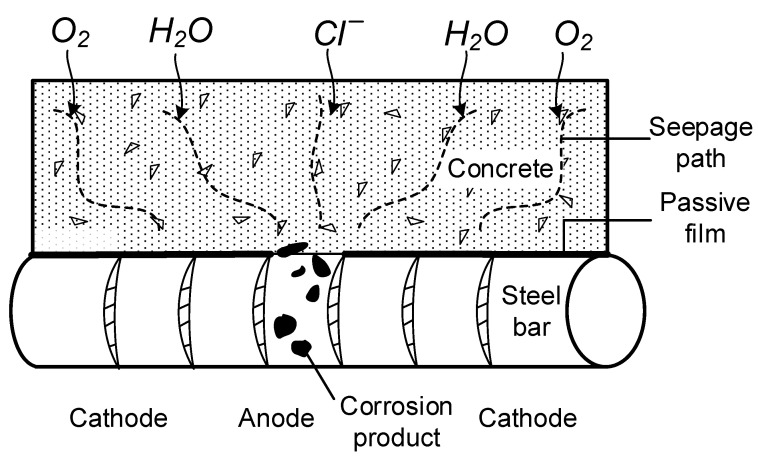
The schematic diagram of electrochemical principle. Adapted with permission from [29].

Electrochemical corrosion and stray current corrosion are two common ways of steel corrosion in reinforced concrete structures. Stray current corrosion needs to meet certain specific conditions to cause steel corrosion, and the steel bars in actual projects are relatively prone to electrochemical corrosion, so electrochemical corrosion has become the main cause of steel corrosion in actual projects, as shown in Figure 4. The reaction formulas for electrochemical corrosion are as follows:

(1) $$4\mathrm{Fe}{\left(\mathrm{OH}\right)}_{2}{+O}_{2}{+2H}_{2}O\to 4\mathrm{Fe}{\left(\mathrm{OH}\right)}_{3}$$

However, the dehydration of Fe(OH)_3_ produces loose and porous corrosion products (Fe_2_O_3_·H_2_O), and under the condition of insufficient oxygen, Fe(OH)_2_ become black Fe_3_O_4_ due to incomplete oxidation. The specific chemical reaction is as follows:

(2) $$6\mathrm{Fe}{\left(\mathrm{OH}\right)}_{2}{+O}_{2}\to {2\mathrm{Fe}}_{3}O_{4}{+6H}_{2}O$$

In this paper, the constant current acceleration method is adopted to carry out corrosion tests on stirrups. The specific corrosion process can be divided into two stages:

#### 2.3.1. The Calculation of Conduction Time

Based on Faraday’s law of electrolysis, the magnitude of current and conduction time can be determined by Equation (3):

(3) Δm=MIt/nF

where Δ*m* is the quality of corrosion products of stirrups; M represents the molar mass of material; I represents the magnitude of constant current; *t* expresses the conduction time; *n* is the number of electrons in the electrodes reflecting the metrological equation; F is Faraday constant of 96,500 C mol^−1^. The diameter of the stirrup is 8 mm and the length of the corroded section is 500 mm or 750 mm. Then, the weight of the stirrups in the corroded section can be calculated to be 197.19 g and 295.79 g, respectively.

For the stirrup with a corrosion rate of 5%, Δ*m*_1_ is 9.86 g and Δ*m*_2_ is 14.79 g. For the stirrup with a corrosion rate of 15%, Δ*m*_1_ is 29.58 g and Δ*m*_2_ is 44.37 g. The molar mass and electrovalence of Fe are 56 kg·mol^−1^ and +2, respectively. The relation of the magnitude of current and conduction time can be obtained through substituting the above parameters into Equation (4):

(4) It=Δm⋅nF/M

When the corrosion rate of stirrups is 5%, I*t*_1_ = 33.98 A·h, I*t*_2_ = 50.97 A·h. When the corrosion rate of stirrups is 15%, I*t*_3_ = 101.95 A·h, I*t*_4_ = 152.92 A·h.

The corrosion current density of stirrups used in this test is 200 μA/cm^2^ and the total surface area of stirrups is A_s1_ = 125.6 mm^2^ and A_s2_ = 188.4 mm^2^, respectively. Therefore, the corrosion current of stirrups is calculated to be 0.25 A and 0.38 A, respectively.

When the magnitude of the current is 0.25 A, the stirrups with the corrosion length of 50 mm need to be electrified for 135.9 h to achieve a corrosion rate of 5%, and 407.8 h to achieve a corrosion rate of 15%. When the current is 0.38 A, for a stirrup with a corrosion length of 75 mm, it takes 134 h to reach a corrosion rate of 5%, and 402.4 h to reach a corrosion rate of 15%.

#### 2.3.2. The Procedure of the Corrosion Experiment

**Figure 5:**
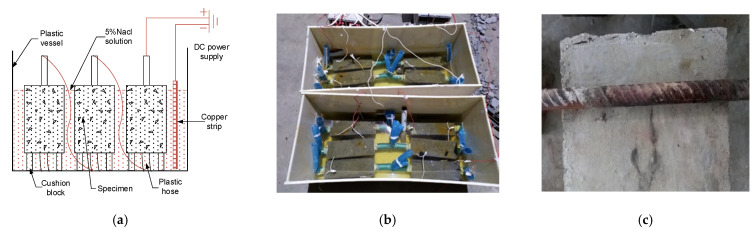
Corrosion method: (**a**) The corrosion system with constant current in laboratory. Adapted with permission from [29]; (**b**) Field experiment; (**c**) Stirrup corrosion.

In the plastic box, the NaCl solution with a mass concentration of 5% is allocated according to the quality of water. Meanwhile, the rusted specimens need to be immersed in the solution for two days in advance. The stirrup is connected to the positive pole of the constant current source and the negative pole of the constant current source is connected to the copper sheet. The current was adjusted to the preset 0.25 A and 0.38 A, and it began to rust. In order to prevent corrosion of the main reinforcement, the surface of the main reinforcement was coated with paint and epoxy resin during the fabrication of the specimen. In addition, rubber mats were placed at the contact points to further prevent the main reinforcement from corrosion at the contact points with the stirrups [8]. For the prevention of water loss, NaCl solution with a mass concentration of 5% was poured into the vessel every day to ensure that the solution exceeded the specimen 5 cm and that the specimen was completely immersed. The corrosion schematic diagram and field picture of this test are shown in Figure 5.

After the final pull-out test, the corroded stirrups were taken out and put into the 5–8% HCl solution reaction to remove the rusting products on the outside surface of the stirrups, and then the corroded products on the outside of the stirrups were removed, wiped, dried, and weighed again. In order to avoid the wire falling off due to rust expansion, a roll of epoxy resin was applied at the connection. The quality of this section was ignored. Therefore, the actual corrosion rate of the stirrup was obtained after the weight of 50 mm length was removed from each stirrup, as shown in Table 2.

### 2.4. Loading Device and Measurement Scheme

**Figure 6:**
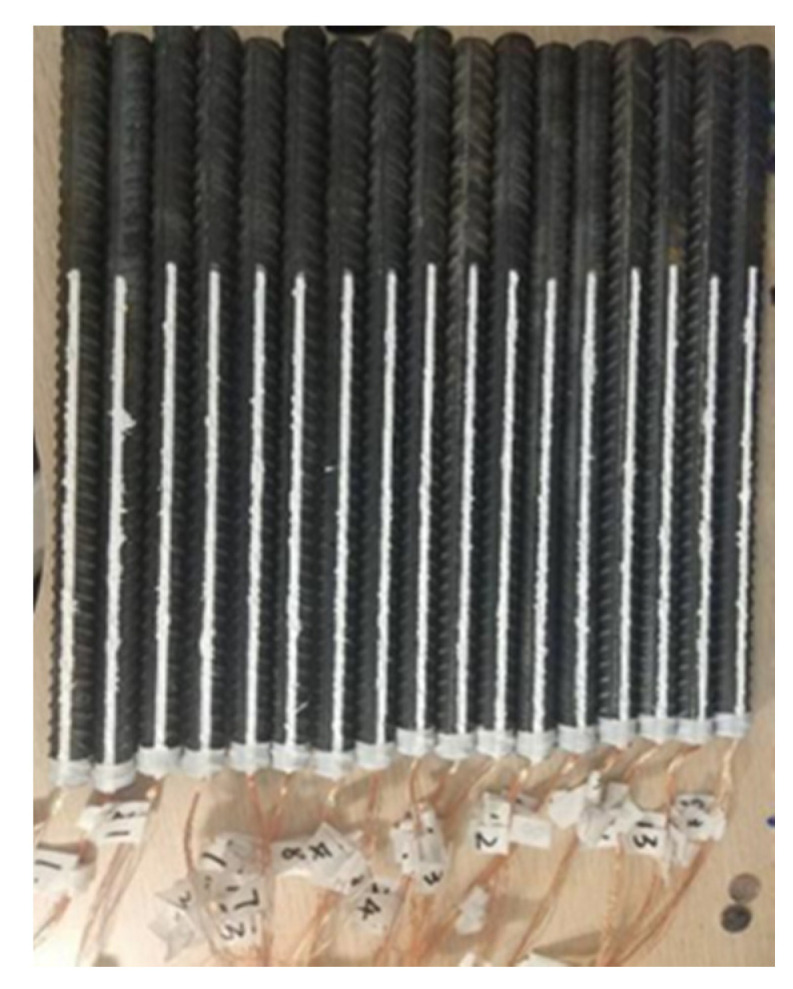
Steel reinforcement: Waterproofing treatment of steel bars. Adapted with permission from [29].

**Figure 7:**
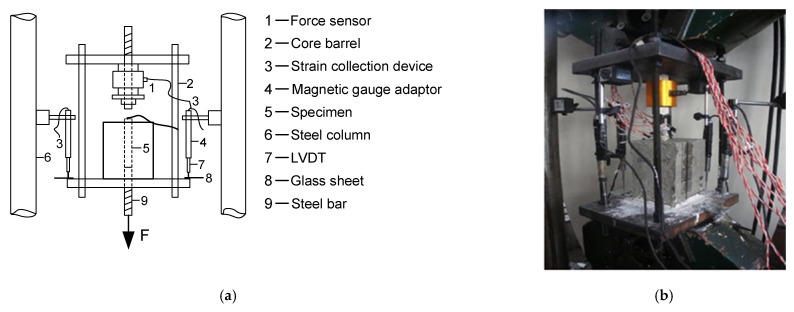
Test loading system: (**a**) The schematic view of loading apparatus Adapted with permission from [29]; (**b**) The loading test in site.

As shown in Figure 6, waterproofing treatment was carried out on the surface of steel bars. Figure 7a,b show the layout of the pulling out test device and force sensor. Two high-precision LVDT displacement meters were used to measure the vertical displacement of the specimen. The slip between reinforcement and concrete is calculated by subtracting the elongation of reinforcement from the total displacement. The *τ*-*s* constitutive relation is obtained by measuring the relationship between the slip of steel bar and load (load–displacement curve) at the loaded end. Preloading is applied to 20 kN, and 60 N/s of force is used to control the speed. Later, in order to obtain the maximum bond stress, the displacement control is adopted with a speed of 0.1 mm/min.

## 3. Experimental Analysis of Bond–Slip Behavior of Reinforced Concrete

### 3.1. Failure Modes of Specimens and Test Data Processing

The equation for the ultimate bond stress (*τ*_u_) is as follows [30]:

(5) $$\tau_{u}=P_{u}/\pi dl$$

where *P*_u_ is the ultimate load of the specimen; *d* is the diameter of longitudinal reinforcement; *l* is the bond length of longitudinal reinforcement in concrete, which is 200 mm.

By observing the performance of 21 specimens in the failure process of this test, it is found that the ultimate failure mode of three groups of specimens without stirrups is splitting failure, while the other specimens with stirrups exhibited pull-out-splitting failure. The specific test data are shown in Table 3.

In this experiment, the pull-out test results after freeze–thaw and stirrup corrosion were analyzed and the effect of freeze–thaw cycles, stirrup corrosion and stirrup spacing on bond performance was studied.

### 3.2. Analysis of Bond Performance Data of Reinforced Concrete

In order to study the effects of three factors on the ultimate slip and bond strength of specimens, the linear fitting of the test results of specimens with two factors consistent and one factor inconsistent is carried out according to the control variable method. The relationship between the ultimate bonding performance and three factors is analyzed. The factors include the corrosion rate of stirrups, the number of freeze-thaw cycles and the stirrup ratio *ρ*_sv_. The *ρ*_sv_ of the specimen can be calculated through Equation (6):

(6) $$\rho_{\mathrm{sv}}=nA_{\mathrm{sv}}/bs$$

where *A*_sv_ is the area of stirrup; the value of *n* is 2; *b* is the width of the specimen, and *s* is stirrup spacing.

Based on the experimental data in Table 4 and Table 5, the influence of stirrup corrosion rate on bonding properties can be revealed by studying the variation of coefficients in these formulas as follows:

When the corrosion rate of the stirrup is low, the corrosion product fills between reinforcement and concrete, and the longitudinal reinforcement is subjected to the annular compressive stress, which increases the friction force between reinforcement and concrete contact surface, thus improving the bonding force. In addition, the small corrosion rate would involve many rust pits on the stirrup surface. When the longitudinal reinforcement and stirrup work together, these rust pits can increase the friction coefficient of the overall concrete [18]. Table 4 and Table 5 reflect the effect of the corrosion rate of stirrups as a single variable on the ultimate bond strength and ultimate slip. When the corrosion rate is not more than 5%, the slope of the expression is positive, indicating that slight corrosion of stirrups can enhance the bond strength of reinforced concrete. When the corrosion rate exceeds 5%, the slopes of the expressions are all negative and the numerical value is large, indicating that as the stirrup corrosion further deepens, the bonding performance of reinforced concrete deteriorates significantly. This law is consistent with the one discovered by Zhou et al. [24], which to some extent validates the accuracy of the experiments conducted in this study.

When the stirrup spacing is set at 150 mm, slight corrosion can significantly improve the bonding performance of specimens frozen and thawed 15 times. However, when the stirrup spacing is set at 100 mm, slight corrosion promotes the bonding performance of specimens without freeze–thaw treatment more obviously. The intercept of the equation represents the original bond strength and the ultimate slip of uncorroded stirrups. By comparing the intercept distance of specimens with different stirrup spacing and freeze–thaw times, it can be found that the increase in the number of freeze–thaw cycles significantly affects the bond strength and ultimate slip between the reinforcement and the concrete, which can be explained by that freeze–thaw cycles can cause micro-cracks to expand within the structure and intersect with the reinforcement. Furthermore, the reduction of the stirrup spacing can increase the bond strength and ultimate slip [15]. This is mainly due to the fact that when the stirrup spacing is small, the densification of the stirrups inhibits the cracking of the concrete and the reduction of the bond strength between the reinforcement and the concrete [31].

**Figure 8:**
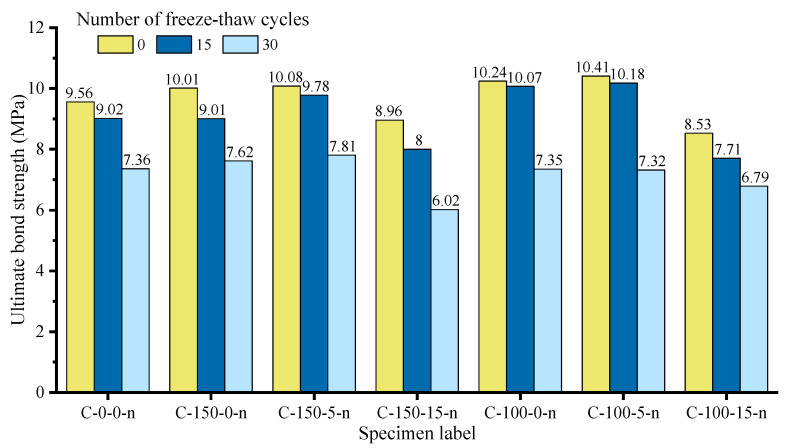
The ultimate bond strength of specimens with different freeze–thaw cycles.

Table 6 reflects the effect of freeze–thaw times as a single variable on ultimate bond strength and ultimate slip. It is obviously seen that the slopes of bond strength and slip are constantly negative, which indicates that the bond performance of reinforced concrete deteriorates gradually with the increase in freeze–thaw times. The ultimate bond stress of specimens with different freeze–thaw cycles is shown in Figure 8. It can be concluded that the ultimate bond strength decreases by 6.1% on average after 15 freeze–thaw cycles, and it decreases by 25.8% on average after 30 freeze–thaw cycles, meaning that when the freeze–thaw cycles are performed 15 times, the bond strength does not decrease significantly, while it decreases rapidly when the cycles are performed 30 times.

**Figure 9:**
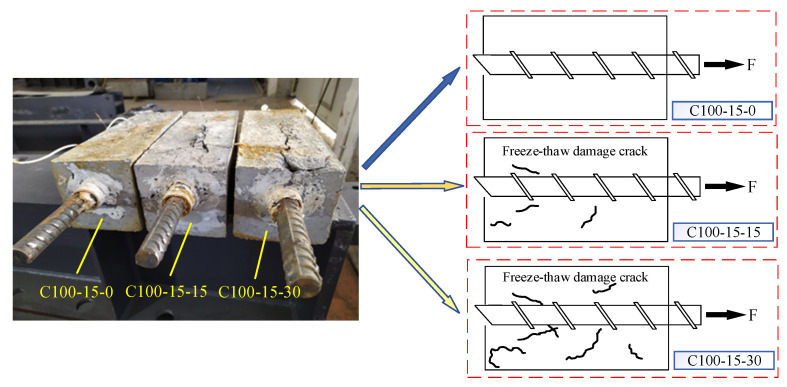
Micro-cracks near steel bars in concrete.

The reduction in bond strength can be explained by the following aspects. The bond strength between reinforcement and concrete is mainly composed of chemical adhesive force, machine bite force and friction force. Repeated freeze–thaw cycles would destroy the chemical adhesive force of the interface between steel bar and concrete. In addition, the repeated freeze–thaw process means that the pore structure in concrete bears repeated fatigue load, which not only leads to the appearance of micro-cracks, but also reduces the strength of concrete and the relative dynamic modulus of elasticity, further resulting in the decrease in bond strength. Furthermore, with the increase in freeze–thaw cycle numbers, micro-cracks continue to extend and eventually intersect with longitudinal reinforcement (e.g., C100-15-n shown in Figure 9), and the confinement effect of concrete on longitudinal reinforcement decreases. At the same time, as the strength of concrete decreases, the circumferential compressive stress of concrete and stirrup on core concrete decreases, which reduces the friction force and mechanical bite force, and further results in a reduction of ultimate bond strength [21].

The arrangement of stirrups would restrain the transverse deformation of concrete during the pull-out of steel bars and this binding force would increase the friction force of concrete on the longitudinal bars and offset the annular tensile stress of the longitudinal bars to the concrete during the tension, thus delaying the crack development. Moreover, the configurations of stirrups could change the constitutive properties of reinforced concrete and improve its tensile strength. According to the data analysis in Table 7, it can be observed that when the stirrup ratio is a single variable, the slopes of the expressions of ultimate bond strength and ultimate slip are positive, so both the ultimate bond strength and ultimate slip increase with the increase in stirrup ratio.

Finally, the range of the coefficients in these formulas with the different corrosion rates, number of freeze–thaw cycles and stirrup ratio are listed in Table 8, so as to sum up the variation of the coefficients and give a more simplified formula. The *X* in equations represents *ω*, *N* and *ρ*_sv_, respectively. The equations fitted by the actual tested data of specimens could contribute to improving the model of the bond between the concrete and steel bar.

### 3.3. Empirical Formula of Ultimate Bond Strength

According to the discussion in the previous section, the thickness of the protective layer and the spacing of stirrups affect the bonding force without considering the external environmental impact. If the bond strength is affected by the external environment, such as chloride ion corrosion, corrosion rate and freeze–thaw cycle times, the empirical formula of bond strength could be referred to as the ultimate strength Equation (7) of steel mixtures proposed by Zhao and Ma [15]:

(7) $$\tau_{u}=\tau_{\mathrm{st}}+\tau_{\mathrm{con}}$$

where *τ*_st_ is the bonding stress of the steel bar, and *τ*_con_ is the bonding stress of concrete.

The data of the above tables are systematically integrated referring to the empirical formula of bond strength, and the deduced formula of bond strength is Equation (8):

(8) $$\tau_{u}=\left(k_{\mathrm{con}}\cdot N+\tau_{0}\right)+\left(A\cdot N+k_{0}\right)\rho_{\mathrm{sv}}$$

In this formula, *k*_con_ = −0.07, *τ*_0_ = 9.75, *A* = 0.94 − 1.84ω, *k*_0_ = 26.81 − 18.24*ω*, where *ω* is the corrosion rate, *N* is the number of freeze–thaw cycles, and *ρ*_sv_ is the stirrup ratio.

The comparison of the measured data of the experiment with the calculation results from the theoretical deduction Equation (8) is shown in Table 9. It can be found that the average error between them is 5.52%, showing that with the consideration of the influence of freeze–thaw cycles, corrosion of stirrups and stirrup ratio, the bond strength derived by the formula is in good agreement with the actual data.

### 3.4. Analysis of Constitutive Model of Bond–Slip Curve

It is found that when the degree of corrosion is low, the ultimate bond strength and ultimate slip slightly increases. As the degree of corrosion continues to increase, the concrete protective layer is cracked. When the corrosion rate is high, as the corrosion rate increases, the amount of slip decreases. First, when the corrosion rate of stirrups is low, the protective layer of concrete has no cracks. As the stirrups rust, their corrosion products fill in the gap between the steel bar and concrete, and as the gap is filled, the corrosion products apply hoop compressive stress to the longitudinal steel bar. The stress increases the friction between the steel bar and the concrete contact surface, thereby increasing the bonding force. Second, when the stirrup corrosion rate is low, there are many rust pits on the surface of the stirrup. When the longitudinal reinforcement and the stirrup work together, these rust pits can increase the coefficient of friction between the reinforcement and the concrete. When the corrosion rate continues to rise, the cracks on the concrete surface continue to develop, the damage to the protective layer is intensified, the hoop compressive stress of the stirrups to the longitudinal reinforcement continues to decrease, and the reduction of friction leads to the decrease in the final bond strength.

**Figure 10:**
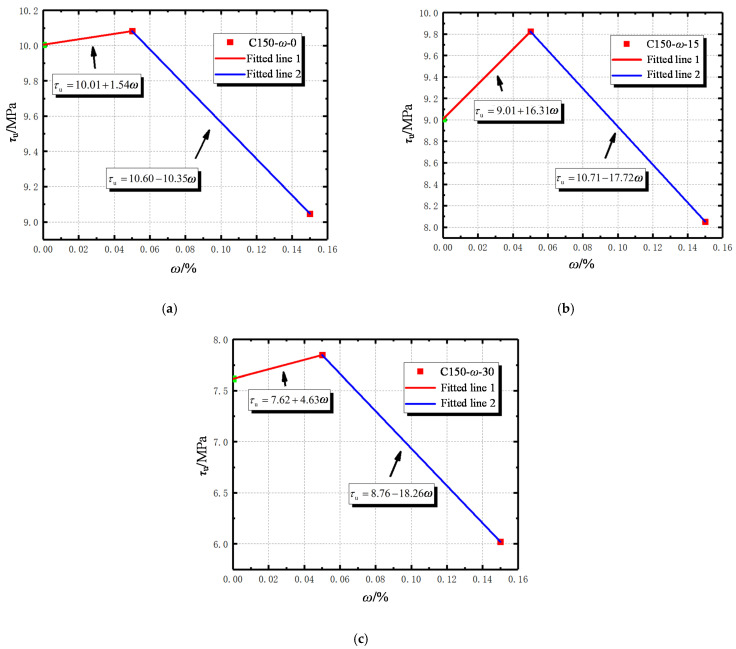
The law of ultimate bond strength changing with rust rate: (**a**) C150-ω-0; (**b**) C150-ω-15; (**c**) C150-ω-30.

The damage of concrete in the repeated freeze–thaw cycles belong to the scope of physical changes. The volume of the small gap in concrete increases when it is refrozen, and cracks begin to appear when reaching the limit of concrete. According to many research results, repeated freeze–thaw cycles are equivalent to the continuous loading and unloading in the concrete interior, and each cycle would cause damage to the durability of the material so that the tiny cracks continue to merge and expand into larger cracks. Taking the specimens with 100 mm stirrup spacing and 5% stirrup corrosion rate as an example, Figure 10 represents *τ*_u_-*S*_u_ diagrams of different freeze–thaw cycle control groups under the same stirrup distribution and stirrup corrosion rate. The initial slope of the rising part of the curve represents the bond stiffness. The steeper the slope of the curve, the greater the bonding stiffness. When the number of freeze–thaw cycles increases from 0 to 15 and further to 30, the diagram becomes smoother and the bond stiffness is smaller, which indicates that freeze–thaw cycles can cause a significant reduction in the durability of concrete.

**Figure 11:**
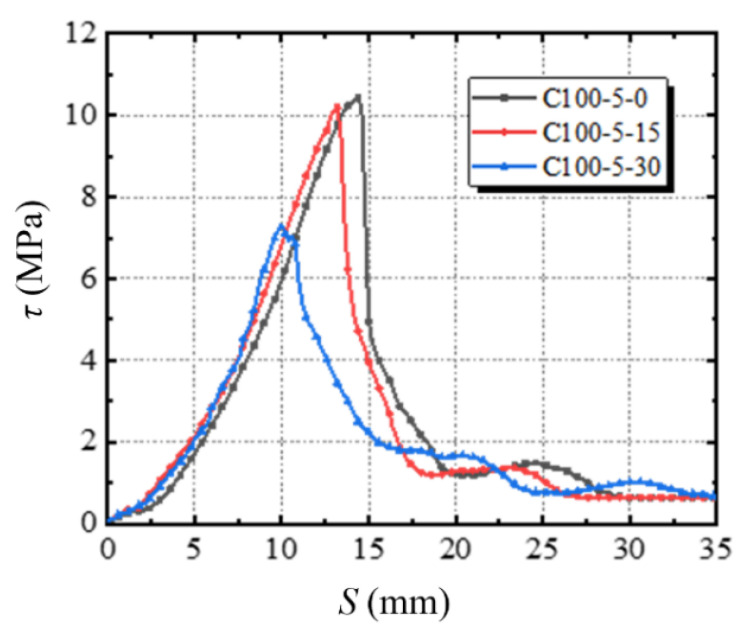
Bond stress–slip curves of reinforcing bars for 0, 15 and 30 freeze–thaw cycles.

Also, it can be seen in Figure 11 that with the same stirrup ratio and corrosion rate, the ultimate bond strength decreases with the increase in freeze-thaw cycle number. Additionally, it can also be seen that with the increase in the number, the coverage area under *τ*-*S* curve is smaller, indicating that the bonding performance deteriorates more, and the energy dissipation capacity and the ductility decrease.

This study combines empirical formulas with experimental results. Based on the model established by the existing scholars [32,33] and combined with the experimental data of this test, a segmented bond–slip model under monotonic loading is established, considering concrete freeze–thaw cycles, stirrup ratio and corrosion rate of stirrups. The entire bond–slip process is divided into three stages: ascending stage, descending stage and residual stress stage. With reference to Figure 11, specific stages are described as follows:

The first stage is the ascending stage, which is usually before the mass emergence of concrete through cracks. At the initial stage of test loading, concrete and longitudinal bars are in a tight bond stage, and the bond–slip curve generally increases linearly, and there is no slip at the free end. As the load continues to increase, the bond–slip curve presents a non-linear growth trend, and the slip increases rapidly. The peak value of bond stress is near the middle and rear parts of the steel bar, and the load will reach its peak soon.

The second stage is the descent stage. At this stage, the bond–slip curve decreases rapidly, and longitudinal cracks appear on the concrete surface. At the same time, the concrete around the longitudinal reinforcement is cracked, and the gripping force, friction force and mechanical bite force rapidly decrease, and the slip increases rapidly.

The third stage is the residual stress stage. Due to the restraint of stirrups, the steel bar pulls out slowly from the concrete, accompanied by a small amount of concrete residue on the surface of the steel bar. The load gradually stabilizes and the displacement slowly increases.

**Figure 12:**
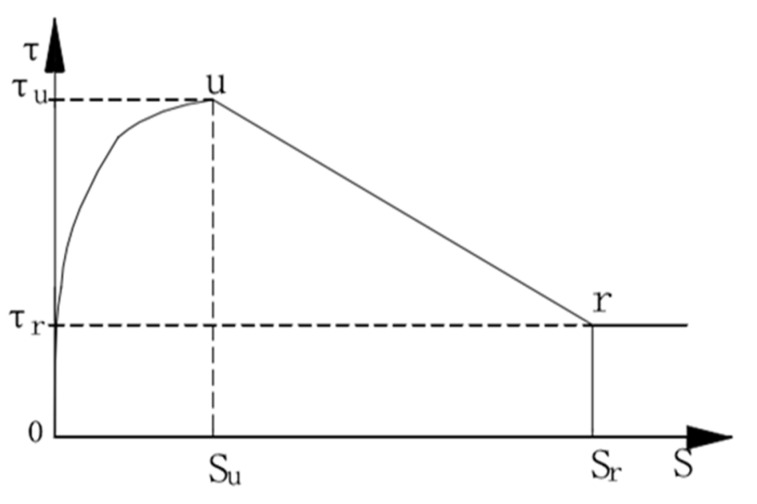
Bond–slip constitutive diagram for reinforced concrete under monotonic load after freeze–thaw cycles.

In summary, the three stages of the bond–slip curve of reinforced concrete are shown in Figure 12.

Ascending stage:

(9) $$\tau =\tau_{u}{\left(\frac{S}{S_{u}}\right)}^{0.4}$$

Descent stage:

(10) $$\tau =\tau_{u}-\left(\tau_{u}-\tau_{r}\right)\left(\frac{S-S_{u}}{S_{r}-S_{u}}\right)$$

Residual stress stage:

(11) $$\tau =\tau_{r}$$

According to Equations (9)–(11), it can be seen that four basic parameters can determine the slip constitutive model so that the process can be described as a whole. The four basic parameters are the ultimate bond stress (*τ*_u_), the ultimate slip (*S*_u_), the residual bond stress (*τ*_r_) and the residual slip (*S*_r_).

The first is the ultimate bond stress (*τ*_u_). *τ*_u_ is derived from the deduced formula of bond strength in Equation (8).

The second is the peak slip value (*S*_u_). According to the fitting results of test data, the ultimate slip of specimens can be calculated by Equation (12):

(12) $$S_{u}=0.75{\left(\frac{c}{d}\right)}^{2}+0.3\rho_{\mathrm{sv}}$$

where *ρ*_sv_ is the stirrup ratio, and *c*/*d* is the ratio of the thickness of the protective layer to the diameter of reinforcement

The third is residual bond stress (*τ*_r_), the value of residual bond stress (*τ*_r_) is proportional to the ultimate bond strength. When the material is fixed, the residual stress is generally a fixed value. Therefore, according to the research of domestic and foreign scholars [29,34,35,36], the functional relationship between the residual stress and the ultimate bond strength is studied. The peak bond stress and residual bond stress are listed in Table 10.

By analyzing the data in Table 10 and taking the average value of *τ*_r_/*τ*_u_ as the reference basis, Equation (13) for calculating the bond strength of residual stress can be obtained:

(13) $$\tau_{r}=0.23\tau_{u}$$

The fourth is residual slip (*S*_r_). According to the test data and the bond–slip curve of the specimens, the ratio of residual slip to ultimate slip is given in Equation (14):

(14) $$S_{r}/S_{u}=a\cdot \rho_{\mathrm{sv}}+b=\left(-86.87\omega -8.31\right)\rho_{\mathrm{sv}}+3.94\omega +1.85$$

In the above, the three stages of the bond–slip constitutive model were analyzed, and the relation formulas of bond and slip were used.

### 3.5. Effect of Anchorage Position on Bond–Slip Constitutive Relation

**Figure 13:**
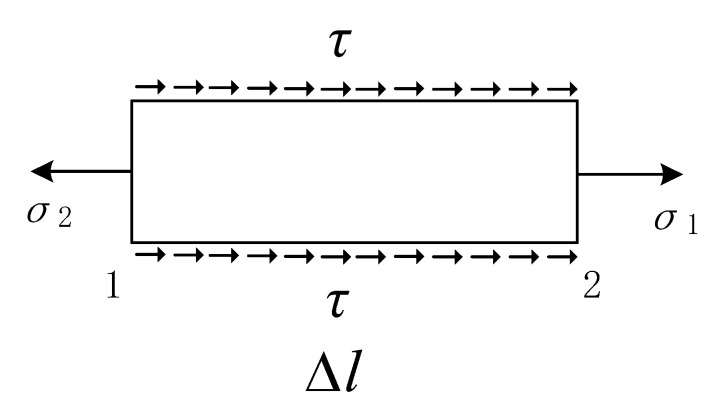
Stress diagram of reinforcement anchorage section.

In order to find a more accurate constitutive model describing the bond stress–strain relationship between reinforcement and concrete, it is necessary to find out the variation of bond strength and ultimate slip of reinforced concrete, caused by different anchorage positions of reinforcement. Methods for calculating different bond stress at different anchorage locations are shown in Figure 13. In the groove of the side ribs of the longitudinal reinforcements, a total of eight steel strain gauges are attached, and the distance between each two steel strain gauges is 25 mm.

Assuming that the strain values measured at points 1 and 2 are *ε*_1_ and *ε*_2_, then *σ*_1_ = E_s_∙*ε*_1_, *σ*_2_ = E_s_∙*ε*_2_. If the cross-sectional area of the reinforcing bar is equal, the calculation is carried out by Equation (15).

(15) $$\tau A=\sigma_{2}A_{s}-\sigma_{1}A_{s}$$

where *A* is the side area of the selected element and *A*_s_ is the cross-section area of the longitudinal bar.

The accumulated total bond stress is different from the average bond stress. According to the principle of reverse distribution, the residual average bond stress is distributed to each section, then the bond stress at each anchorage position can be obtained and the bond stress curve can be drawn.

**Figure 14:**
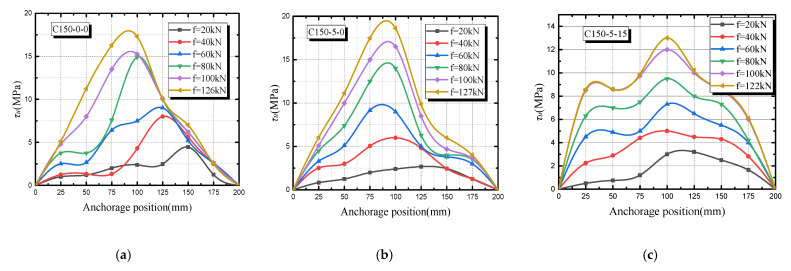
Bond stress-anchorage position relationship: (**a**) C150-0-0; (**b**) C150-5-0; (**c**) C150-5-15.

Since some specimens are in the repeated freeze–thaw cycles, the temperature is too low, which causes water to enter the specimens and leads to the failure of the strain gauge. Therefore, the bond–slip curve of the anchorage section is mainly composed of three specimens: C150-0-0, C150-5-0 and C150-5-15. According to Figure 13, bond stress and slip position have the following rules:

1. With the change of anchorage position, the bond stress on the longitudinal reinforcement can be regarded as a parabola. The bond stress in the middle is the largest, while the stress at the loading end and the free end is the smallest.
2. When the tension is 20 kN, the peak value of bond stress is near the loading end, and most of the stress is balanced at the loading end. Therefore, the bond stress near the free end gradually decreases.
3. As the load increases, the stress at each anchorage section increases. When the adhesion force at the loading end cannot be balanced, a local tension crack appears with local slip. It can be seen that the peak value of bonding force is generally 0.6*l*_a_ away from the loading end.
4. When the bonding force at the free end cannot offset the external load, cracks appear at the entire contact surface of the reinforcing steel and concrete simultaneously. The bond stress at each point decreases rapidly, and the steel bar is pulled out slowly.
5. Comparing Figure 14a,b, it can be observed that when the stirrup corrosion rate is low, the peak value is enhanced and the stirrup rusts slightly, increasing the restraint effect of concrete on the longitudinal reinforcement. Comparing Figure 14b,c, it can be found that with the increase in the number of freeze–thaw cycles, the curve becomes steeper and the peak value further decreases.

## 4. Simulation of Reinforced Concrete Pull-Out Specimen

### 4.1. Establishment of Finite Element Model and Selection of Elements

**Figure 15:**
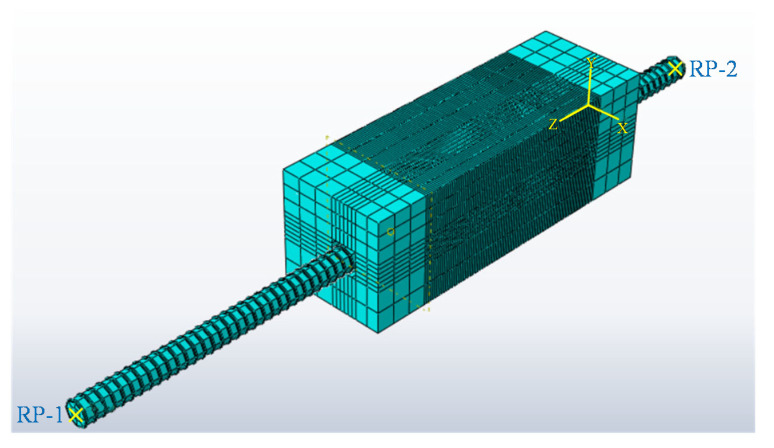
Schematic diagram of three-dimensional model of finite element analysis.

The purpose of this simulation is to analyze the parameters, quality standards and mechanical properties of the eccentrically drawn specimens. Based on the actual conditions, an accurate three-dimensional model is constructed in ABAQUS to simulate the whole process of steel bar pulling out from concrete step by step. The uniaxial tensile stress and compressive stress constitutive models of concrete [37] are used in three-dimensional modeling. Since the freeze–thaw cycles are considered in this experiment, the damage constitutive relationship of concrete under freeze–thaw cycles is quoted from Guangcheng Long [38]. Figure 15 shows the finite element model of bond–slip in reinforced concrete established by ABAQUS software. In this paper, the solid element is used to simulate the stress conditions in the extraction process. Because the longitudinal reinforcement is completely wrapped by concrete, the longitudinal reinforcement element adopts the C3D10 type. The stirrups have little influence on the strength and the protective layer is thin. In order to reduce the amount of calculation, the stirrups are simplified as truss elements, and the element is T3D2 type. The concrete element within 50 mm before and after stirrup is C3D10 type, and the element beyond this range is C3D8R type. Spring2 double-spring coupling unit is employed to simulate the bond–slip between the reinforcement and concrete. Specifically, the vertical spring simulates the normal compression of the reinforcement by the concrete and can be treated as a rigid coupling. The horizontal spring simulates the tangential bond between the reinforcement and the concrete, and its value is determined by the bond–slip curve shown in Figure 12.

### 4.2. Verification of the FEA Model

The ABAQUS data of 21 eccentrically tensioned specimens were analyzed, and the simulated values were compared with the bond strength of the test. The comparison data are shown in Table 11. As shown in Table 11, the average error between the ultimate pulling load obtained from the calculation of ABAQUS and the actual value obtained from the test is only 4.32%, so the two results are very close. The average error between the test data and results based on the formula is only 5.52%, which just verifies the rationality and correctness of the experimental data in this study. Therefore, the ABAQUS finite element analysis method proposed in this section is accurate and reliable.

### 4.3. Finite Element Stress Distribution Analysis

**Figure 16:**
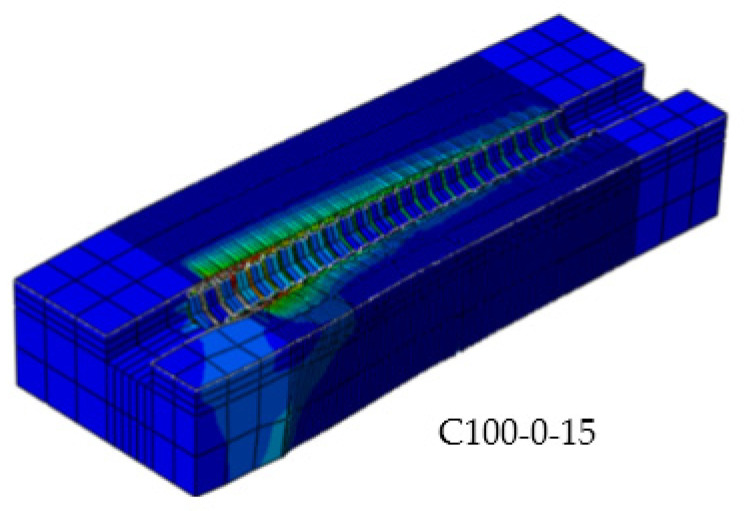
Von Mises stress distribution in concrete simulated by finite element method.

Finite element analysis can be used to get the stress visualization for steel reinforcement, stirrup and concrete at any position during the pull-out test, as well as the development process of concrete crack, thus making up for the defect of the actual test. There are 21 groups of control experiments in this simulation. The stress contours of two key components of a representative specimen are listed. The von Mises stress cloud diagram of the concrete under the ultimate load is shown in Figure 16. It can be seen from Figure 16 that in the process of pull-out, the stress concentration is at the loading end of the specimen, where the concrete will quickly crack and break. Cracks occur when the bond tensile stress between concrete and steel bar exceeds the tensile strength of concrete.

**Figure 17:**
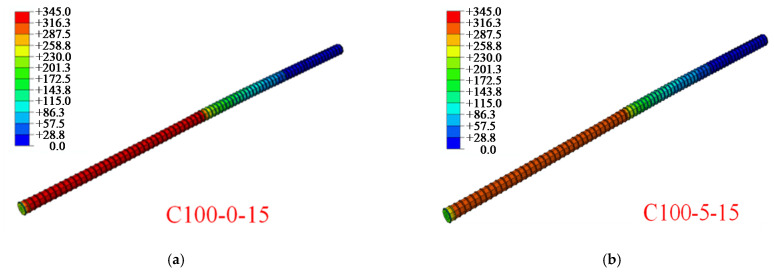

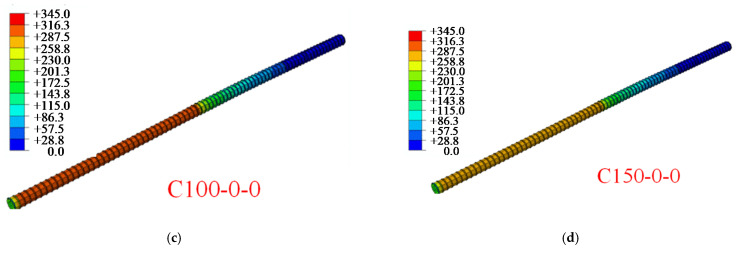
Von Mises stress distribution in reinforcement simulated by finite element method (MPa): (**a**) C100-0-15; (**b**) C100-5-15; (**c**) C100-0-0; (**d**) C150-0-0.

Figure 17 displays the von Mises stress cloud diagram of the reinforcement under the ultimate load. The meaning for red in the figure is that the steel bar has reached the ultimate tensile strength. In the simulation process, only the elastic part is selected. The maximum stress of reinforcing bar usually appears at 10–12 stirrup casing, that is, in the middle and back of anchorage position, which is consistent with the anchorage position measured by strain gauges.

### 4.4. Comparative Analysis of Simulated Bond–Slip Diagrams

**Figure 18:**
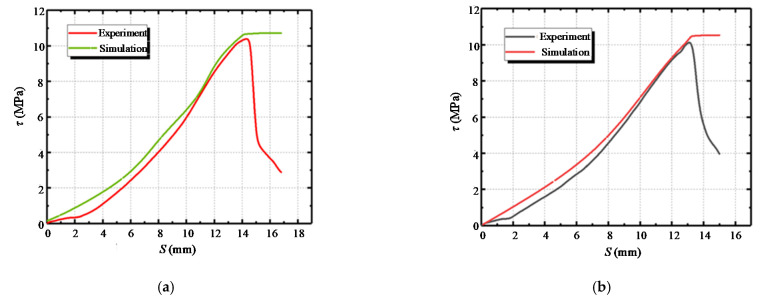

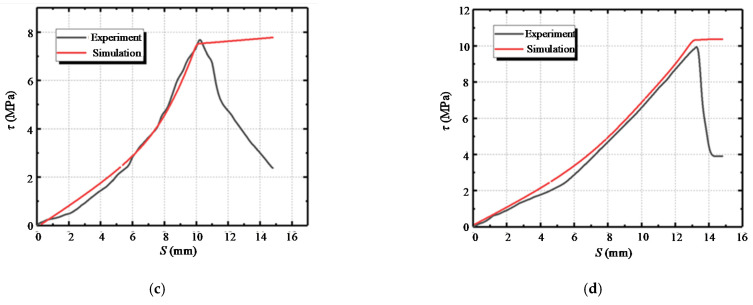
Comparison of bond–slip diagrams for the experiment and simulation: (**a**) C100-5-0; (**b**) C100-5-15; (**c**) C100-5-30; (**d**) C150-5-0.

The comparison of the experimental and simulation results of the bond–slip diagrams for specimens with different numbers of freeze–thaw cycles is shown in Figure 18. It can be found that the accurate calculation through simulation coincides with the rising section of the measured data, but a certain gap is reflected in the downward trend. It can also be seen that the simulated *τ*-*S* diagram has only a rising section, while the measured curve has both ascending and descending segments. This is mainly due to that the ascending segment of the curve is of more research interest, while the simulation of the descending segment is not of much significance. In the later stage, the true force and displacement relationship can also be brought in, and the descending section can be drawn, but it is of little significance. The ultimate bond strength mainly guides most of the experimental research.

## 5. Conclusions

In this paper, the effects of concrete freeze–thaw cycles, stirrup corrosion and stirrup spacing on structural bond strength are investigated based on the eccentric pull-out test of reinforced concrete. A special model is established by using ABAQUS finite element software. The main conclusions are as follows:

1. Comparing the failure modes of different specimens, it can be seen that stirrup spacing, corrosion rate, and the number of freeze–thaw cycles have an effect on the failure mode of the structure in the monotonic pull-out test. When the stirrups are not deployed, the mode of concrete destruction is splitting, and when the stirrups are placed, the mode of concrete destruction is pull-out.
2. Slight corrosion of the stirrups will increase the circumferential compressive stresses, which increases the bonding force and slip. The bond strength and ultimate slip increase linearly with the decrease in stirrup spacing. The freeze–thaw cycles reduce the ultimate bond strength and slip, and the larger the number of freeze–thaw cycles, the more obvious the strength decrease. The stress at the anchorage position increases with the increase in external load and presents a parabolic distribution. The peak value of bond stress at the initial stage of loading is close to the loading end, and 0.6*l*_a_ from the free end at the final stage.
3. The empirical formula of ultimate bond strength under the coupling action of stirrup corrosion rate, freeze–thaw cycles and stirrup ratio was established. The average error between the calculation results of the formula and the experimental data is 5.52%, which proves the reliability of the empirical formula of ultimate bond strength. In addition, a bond–slip constitute model was established and the coefficients in the formulas were determined.
4. An accurate three-dimensional model was established based on ABAQUS, which took into account the freeze–thaw cycles and stirrups and progressively simulated the whole process of the reinforcement pulling out of the concrete. The actual value of the test was compared with the ultimate pull-out load obtained by the simulation, and the average error is 4.32%, which verifies the rationality and correctness of the test data. Therefore, the bond–slip constitutive model established in this paper is generalizable and could provide a reference for further finite element analysis.

## Figures and Tables

**Table 1:** Corrosion grade of stirrups, stirrup spacing and freeze–thaw cycles of tested specimens.

Specimen Label	Stirrup Spacing (mm)	Stirrup Corrosion Rate (%)	Freeze–Thaw Cycle Number	Specimen Label	Stirrup Spacing (mm)	Stirrup Corrosion Rate (%)	Freeze–Thaw Cycle Number
C0-0-0	0	0	0	C100-15-30	100	15	30
C0-0-15	0	0	15	C150-0-0	150	0	0
C0-0-30	0	0	30	C150-0-15	150	0	15
C100-0-0	100	0	0	C150-0-30	150	0	30
C100-0-15	100	0	15	C150-5-0	150	5	0
C100-0-30	100	0	30	C150-5-15	150	5	15
C100-5-0	100	5	0	C150-5-30	150	5	30
C100-5-15	100	5	15	C150-15-0	150	15	0
C100-5-30	100	5	30	C150-15-15	150	15	15
C100-15-0	100	15	0	C150-15-30	150	15	30
C100-15-15	100	15	15	/	/	/	/

**Table 2:** The measured corrosion rate of steel bar.

Conduction Time (h)	Original Weight (g)	Post Reaction Weight (g)	Target Corrosion Rate	Actual Corrosion Rate	Average Corrosion Rate
135.92	169	161.01	5%	4.73%	5.09%
134.13	253.5	239.71	5%	5.44%	
407.8	169	142.25	15%	15.83%	15.94%
402.42	253.5	212.84	15%	16.04%	

**Table 3:** Test data of ultimate bond strength *τ*_u_ and ultimate slip *S*_u_.

Specimen Label	*P*_u_ (kN)	*S*_u_ (mm)	*τ*_u_ (MPa)	Specimen Label	*P*_u_ (kN)	*S*_u_ (mm)	*τ*_u_ (MPa)
C0-0-0	120.19	11.80	9.56	C150-15-30	75.67	8.77	6.02
C0-0-15	113.35	10.00	9.02	C100-0-0	128.64	13.40	10.24
C0-0-30	92.55	10.05	7.36	C100-0-15	126.52	12.80	10.07
C150-0-0	125.73	12.70	10.01	C100-0-30	92.39	10.03	7.35
C150-0-15	113.2	11.41	9.01	C100-5-0	130.82	14.40	10.41
C150-0-30	95.71	9.43	7.62	C100-5-15	127.97	13.20	10.18
C150-5-0	126.7	13.20	10.08	C100-5-30	91.98	9.95	7.32
C150-5-15	122.85	12.54	9.78	C100-15-0	107.15	12.10	8.53
C150-5-30	98.09	10.01	7.81	C100-15-15	96.93	11.03	7.71
C150-15-0	112.62	11.60	8.96	C100-15-30	85.27	9.16	6.79
C150-15-15	100.52	10.80	8.00	/	/	/	/

**Table 4:** The ultimate bond strength as a function of stirrup corrosion rate *ω*.

First Stage (0 ≤ ω ≤ 5%)			Second Stage (5 < ω ≤ 15%)		
Equation	$\tau_{u1}=a_{r1}\cdot \omega +b_{r1}$		Equation	$\tau_{u2}=a_{r2}\cdot \omega +b_{r2}$	
Specimen Label	*a*_r1_	*b*_r1_	Specimen Label	*a*_r2_	*b*_r2_
C150-ω-0	1.54	10.01	C150-ω-0	−10.35	10.60
C150-ω-15	16.31	9.01	C150-ω-15	−17.72	10.71
C150-ω-30	4.63	7.62	C150-ω-30	−18.26	8.76
C100-ω-0	3.47	10.24	C100-ω-0	−17.89	11.30
C100-ω-15	2.31	10.07	C100-ω-15	−24.70	11.42
C100-ω-30	8.96	7.35	C100-ω-30	−10.15	8.31

**Table 5:** The ultimate slip as a function of stirrup corrosion rate *ω*.

First Stage (0 ≤ ω ≤ 5%)			Second Stage (5% < ω ≤ 15%)		
Equation	$S_{u1}=a_{s1}\cdot \omega +b_{s1}$		Equation	$S_{u2}=a_{s2}\cdot \omega +b_{s2}$	
Specimen Label	*a*_s1_	*b*_s1_	Specimen Label	*a*_s2_	*b*_s2_
C150-ω-0	10.00	12.70	C150-ω-0	−16	14
C150-ω-15	22.6	11.41	C150-ω-15	−17.4	13.41
C150-ω-30	11.6	9.43	C150-ω-30	−12.4	10.63
C100-ω-0	20	13.4	C100-ω-0	−23	15.55
C100-ω-15	8	12.8	C100-ω-15	−21.7	14.29
C100-ω-30	1.61	9.85	C100-ω-30	−8.72	10.47

**Table 6:** The ultimate bond strength and ultimate slip as a function of freeze–thaw cycle number *N*.

Ultimate Bond Strength (*N* = 0, 15, 30)			Ultimate Slip (*N* = 0, 15, 30)		
Equation	$\tau_{u}=a_{r}\cdot N+b_{r}$		Equation	$S_{u}=a_{s}\cdot N+b_{s}$	
Specimen Label	*a*_r_	*b*_r_	Specimen Label	*a*_s_	*b*_s_
C150-0-N	−0.080	10.07	C150-0-N	−0.110	12.82
C150-5-N	−0.074	10.37	C150-5-N	−0.110	13.51
C150-15-N	−0.100	9.20	C150-15-N	−0.090	11.81
C100-0-N	−0.096	10.66	C100-0-N	−0.120	13.78
C100-5-N	−0.087	10.77	C100-5-N	−0.150	14.73
C100-15-N	−0.060	8.62	C100-15-N	−0.100	12.23

**Table 7:** The ultimate bond strength and ultimate slip as a function of stirrup ratio *ρ*_sv_.

Ultimate Bond Strength			Ultimate Slip		
Equation	$\tau_{u}=a_{r}\cdot \rho_{\mathrm{sv}}+b_{r}$		Equation	$S_{u}=a_{s}\cdot \rho_{\mathrm{sv}}+b_{s}$	
Specimen Label	*a*_r_	*b*_r_	Specimen Label	*a*_s_	*b*_s_
$C\rho_{\mathrm{sv}}-0-0$	16.69	9.56	$C\rho_{\mathrm{sv}}-0-0$	38.91	11.76
$C\rho_{\mathrm{sv}}-0-15$	23.11	8.90	$C\rho_{\mathrm{sv}}-0-15$	67.22	9.90
$C\rho_{\mathrm{sv}}-0-30$	22.39	7.28	$C\rho_{\mathrm{sv}}-0-30$	32.94	9.36

**Table 8:** The unified empirical formula.

Equation	$\tau_{u}=a_{\tau }\cdot X+b_{\tau }$		$S_{u}=a_{s}\cdot X+b_{s}$	
*X*	*a_τ_*	*b_τ_*	*a*_s_	*b*_s_
Corrosion rate (*ω*)	$1.54\leq a_{\tau 1}\leq 16.31$	$7.35\leq b_{\tau 1}\leq 10.24$	$1.61\leq a_{s1}\leq 22.6$	$9.43\leq b_{s1}\leq 13.4$
	$-24.70\leq a_{\tau 2}\leq -10.15$	$8.31\leq b_{\tau 2}\leq 10.42$	$-21.7\leq a_{s2}\leq -8.72$	$10.47\leq b_{s2}\leq 15.55$
Freeze–thaw cycles (*N*)	$-0.100\leq a_{\tau }\leq -0.060$	$8.62\leq b_{\tau }\leq 10.77$	$-0.150\leq a_{s}\leq 0.090$	$11.81\leq b_{s}\leq 14.73$
Stirrup ratio (*ρ*_sv_)	$16.69\leq a_{\tau }\leq 23.11$	$7.28\leq b_{\tau }\leq 9.56$	$32.94\leq a_{s}\leq 67.22$	$9.36\leq b_{s}\leq 11.76$

**Table 9:** Comparison of ultimate bond strength between experimental measurement and formula derivation.

Specimen Label	Test Values (MPa)	Predicted Value (MPa)	Error	Specimen Label	Test Values (MPa)	Predicted Value (MPa)	Error
C0-0-0	9.56	9.75	1.99%	C150-15-30	6.02	6.52	8.31%
C0-0-15	9.02	8.7	3.55%	C100-0-0	10.24	10.83	5.76%
C0-0-30	7.36	7.65	3.94%	C100-0-15	10.07	10.34	2.68%
C150-0-0	10.01	10.47	4.60%	C100-0-30	7.35	7.86	6.94%
C150-0-15	9.01	9.8	8.77%	C100-5-0	10.41	10.83	4.03%
C150-0-30	7.62	8.12	6.56%	C100-5-15	10.18	9.73	4.42%
C150-5-0	10.08	10.47	3.87%	C100-5-30	7.32	8.11	10.79%
C150-5-15	9.78	9.42	3.68%	C100-15-0	8.53	9.41	10.32%
C150-5-30	7.81	8.02	2.69%	C100-15-15	7.71	8.01	3.89%
C150-15-0	8.96	9.4	4.91%	C100-15-30	6.79	7.24	6.63%
C150-15-15	8.00	8.61	7.62%	/	/	/	/

**Table 10:** The peak bond stress and residual bond stress.

Specimen Label	*τ_u_* (MPa)	*τ_r_* (MPa)	*τ*_r_/*τ*_u_	Specimen Label	*τ_u_* (MPa)	*τ_r_* (MPa)	*τ*_r_/*τ*_u_
C100-0-0	10.24	2.15	0.21	C150-0-0	10.01	2.01	0.20
C100-0-15	10.07	2.14	0.21	C150-0-15	9.01	1.78	0.20
C100-0-30	7.35	1.80	0.24	C150-0-30	7.62	1.64	0.22
C100-5-0	10.41	2.25	0.22	C150-5-0	10.08	2.02	0.20
C100-5-15	10.18	2.10	0.21	C150-5-15	9.78	1.80	0.18
C100-5-30	7.32	1.63	0.22	C150-5-30	7.81	1.62	0.21
C100-15-0	8.53	1.97	0.23	C150-15-0	8.96	1.78	0.20
C100-15-15	7.71	1.71	0.22	C150-15-15	8.00	1.73	0.22
C100-15-30	6.79	1.54	0.23	C150-15-30	6.02	1.50	0.25

**Table 11:** Comparison of simulation and test results.

Specimen Label	Test Values (MPa)	Simulation Values (MPa)	Error	Specimen Label	Test Values (MPa)	Simulation Values (MPa)	Error
C0-0-0	9.56	9.98	4.39%	C150-15-30	6.02	5.83	3.16%
C0-0-15	9.02	9.34	3.55%	C100-0-0	10.24	10.73	4.79%
C0-0-30	7.36	8.03	9.10%	C100-0-15	10.07	10.35	2.78%
C150-0-0	10.01	10.29	2.80%	C100-0-30	7.35	7.12	3.13%
C150-0-15	9.01	9.57	6.22%	C100-5-0	10.41	10.66	2.40%
C150-0-30	7.62	7.02	7.87%	C100-5-15	10.18	10.48	2.95%
C150-5-0	10.08	10.31	2.28%	C100-5-30	7.32	7.51	2.60%
C150-5-15	9.78	10.11	3.37%	C100-15-0	8.53	8.42	1.29%
C150-5-30	7.81	7.41	5.12%	C100-15-15	7.71	7.15	7.26%
C150-15-0	8.96	9.62	7.37%	C100-15-30	6.79	6.52	3.98%
C150-15-15	8.00	8.34	4.25%	/	/	/	/

## Data Availability

Data are contained within the article.
